# Quinine—When to Give It and How to Give It

**Published:** 1887-10

**Authors:** L. B. Banchelle

**Affiliations:** Thomasville, Ga.


### QUININE—WHEN TO GIVE IT AND HOW TO GIVE IT.

BY L. B. BANCHELLE, M. D., THOMASVILLE, GA.

Since the days of the Countess of Cinchon this drug has been known to the profession. The indications for its administration are very numerous, and it is at times greatly abused as well. In the early days of its history it was regarded as a drug of such power that it was exhibited in very diminutive doses. And, too, the diseases in which it was regarded useful were quite limited in number. It is altogether the order of the day for one extreme to follow another in many things—so in this. From Homæopathic doses it has become quite "the go" to "pour it in bountifully." Then, too, the latitude of its use has greatly increased. While progress is well and wise, sometimes we have the termity to proceed too far. A daring M. D. a few years ago hired a freedman to take an ounce at once to observe its effects. They were paralysis of the optic nerves for a time. Pretty bad. Twice within a few years the writer has been called in consultation where similar effects followed its injudicious exhibition in private practice. From too little to too much—extremes should be shunned.

In what diseases is it indicated? That is what we wish to know. In all febrile affections, whether idiopathic or traumatic. What! in typhus and typhoid fevers? Yes, it is as efficacious in these as any other variety of fever. It chould be combined with whatever remedies that the various complications of the disease demands. It combines well with bismuth in bowel complications and with the various opiates in cephalic complications. What time in the twenty-four hours should it be given in any disease? With *rare exceptions*, *only in the forenoon!* Why? Because all fevers run to ebb tide, from 3 o'clock a.m. till 12 m. Then, too, it is a nervous excitant, and its effects should wear off before nature's time to sleep comes. Suppose the fever subsides at any time between m., or *high twelve* and *low twelve*, should it be given during the night? *No.* Sleep is *sick* as well as "*weary* nature's sweet restorer," and he who disturbs the nightly slumbers of the sick proves himself to be a hurtful "intermeddler in other men's matters." What about heroic doses? Twenty grains are sufficient in any case, at one dose, in extreme cases. How often should it be repeated? Once in two hours. How many doses in a day? If properly adapted, in size and nature of the disease, *three only.*

Is it indicated in any class of neuralgias? *Rarely.* What, then, must we do for neuralgia? *Remove the cause*; then, with a suitable anodyne, put the nervous system to sleep till it forgets its insubordinate habit. Is it good for rheumatism? Yes. How so? Because there is *fever*, and, too, it helps in its influence upon the nervous system. Will it not do the same in neuralgia? Possibly; but its effects are only paliative. Are they not the same in rheumatism? Measurably; but, in the case of neuralgia, the cause is neither occult or immovable. In rheumatism, the cause is *very*

*often* both hidden and immovable, for quite a time—often a *life time*, and then transmitted to succeeding generations.

Is quinine a specific for any disease? *Per se*, no. It is only a rivulet or brook, that combines with other remedies to help nature to throw off its load of disease. Will it ever be displaced with other, more efficient, remedies? Who can tell? The class of febrile antis is on the increase. The chemists' laboratories are at work, bringing from their hidden recesses many new things.

Who makes the best article of quinine? No one knows. With a large experience, and a daily use of the drug in many diseases, with all the "makes," I find just no difference. If removed, forgotten, the supply exhausted, or it numbered with the lost arts, where can we find the next best febrifuge? In antipyrin and antifibrin, and, perhaps, in ipecacuanha. Of this drug, perhaps, more another time.

